# Engineering proton-coupled hexose uptake in *Saccharomyces cerevisiae* for improved ethanol yield

**DOI:** 10.1186/s13068-022-02145-7

**Published:** 2022-05-07

**Authors:** Sophie C. de Valk, Susan E. Bouwmeester, Erik de Hulster, Robert Mans

**Affiliations:** Department of Biotechnology, Delft University of Technology, Van der Maasweg 9, 2629HZ Delft, The Netherlands

**Keywords:** Bioethanol, Sugar transport, Yeast physiology, Energy metabolism, Evolutionary engineering

## Abstract

**Background:**

In the yeast *Saccharomyces cerevisiae*, which is widely applied for industrial bioethanol production, uptake of hexoses is mediated by transporters with a facilitated diffusion mechanism. In anaerobic cultures, a higher ethanol yield can be achieved when transport of hexoses is proton-coupled, because of the lower net ATP yield of sugar dissimilation. In this study, the facilitated diffusion transport system for hexose sugars of *S. cerevisiae* was replaced by hexose–proton symport.

**Results:**

Introduction of heterologous glucose– or fructose–proton symporters in an *hxt*^*0*^ yeast background strain (derived from CEN.PK2-1C) restored growth on the corresponding sugar under aerobic conditions. After applying an evolutionary engineering strategy to enable anaerobic growth, the hexose–proton symporter-expressing strains were grown in anaerobic, hexose-limited chemostats on synthetic defined medium, which showed that the biomass yield of the resulting strains was decreased by 44.0-47.6%, whereas the ethanol yield had increased by up to 17.2% (from 1.51 to 1.77 mol mol hexose^−1^) compared to an isogenic strain expressing the hexose uniporter *HXT5*. To apply this strategy to increase the ethanol yield on sucrose, we constructed a platform strain in which all genes encoding hexose transporters, disaccharide transporters and disaccharide hydrolases were deleted, after which a combination of a glucose–proton symporter, fructose–proton symporter and extracellular invertase (*SUC2*) were introduced. After evolution, the resulting strain exhibited a 16.6% increased anaerobic ethanol yield (from 1.51 to 1.76 mol mol hexose equivalent^−1^) and 46.6% decreased biomass yield on sucrose.

**Conclusions:**

This study provides a proof-of-concept for the replacement of the endogenous hexose transporters of *S. cerevisiae* by hexose-proton symport, and the concomitant decrease in ATP yield, to greatly improve the anaerobic yield of ethanol on sugar. Moreover, the sugar-negative platform strain constructed in this study acts as a valuable starting point for future studies on sugar transport or development of cell factories requiring specific sugar transport mechanisms.

**Supplementary Information:**

The online version contains supplementary material available at 10.1186/s13068-022-02145-7.

## Background

The yeast *Saccharomyces cerevisiae* is well known as an excellent producer of ethanol, and has therefore been widely applied to produce alcoholic beverages, as well as for the industrial-scale production of bioethanol [1, 2]. Even under aerobic conditions, this yeast preferentially dissimilates glucose via alcoholic fermentation when glucose is present in sufficiently high concentrations (> 1 g L^−1^, Crabtree effect), which is remarkable since respiration yields substantially more ATP per molecule of dissimilated glucose [3, 4]. Different hypotheses have been proposed to explain the occurrence of such ‘inefficient’ modes of substrate dissimilation, but to date this topic is still subject to discussion [5–8]. The emergence of Crabtree-positive yeasts 100–150 million years ago probably coincided with the emergence of fruit-bearing plants, providing a sugar-rich niche in which these yeasts evolved [9, 10]. In such environments, the high extracellular glucose concentration can drive the uptake of glucose by yeast cells via diffusion, which is exemplified by the occurrence of many hexose transporters with a facilitated diffusion mechanism in *S. cerevisiae*. In total, a set of 20 hexose transporters has been described in this yeast with various kinetic properties, whose regulation allows for fast uptake of glucose at high (> 20 g/L) to near-zero glucose concentrations [11–14]. To facilitate uptake and conversion of glucose at low extracellular concentrations, activity of high-affinity transporters (*K*_M_~ 1 mM) is required, in combination with hexokinase activity to ‘trap’ the sugar inside the cell in its phosphorylated form and therefore maintain a glucose concentration gradient over the cellular membrane [15]. There are other yeast species that, on the contrary, express hexose–proton symporters that make use of the proton motive force to drive transport of sugar into the cells, which can give a selective advantage when environmental hexose concentrations are very low (*K*_M_ 20–200 µM) [16–19]. The proton motive force is maintained by export of protons by a plasma membrane H^+^-ATPase, and therefore this sugar transport system requires net investment of ATP for its activity [20]. Thus, the transport mechanism of sugar uptake can have a substantial effect on the overall efficiency of sugar dissimilation in terms of ATP yield, especially when the substrate is dissimilated via fermentation, which results in a low amount of ATP produced per mol of substrate.

This concept and its effect on yeast physiology and bioethanol production was previously demonstrated for dissimilation of the disaccharide sucrose in *S. cerevisiae* [21]. In wild-type *S. cerevisiae*, sucrose is mainly hydrolyzed outside of the cell by the invertase Suc2, after which the resulting monosaccharides are taken up by facilitated diffusion [11, 22, 23]. However, when the extracellular invertase activity was diminished, sucrose was taken up by disaccharide transporters, which employ a proton symport mechanism [24]. In *S. cerevisiae*, export of a proton via the plasma membrane ATPase Pma1 comes at the cost of 1 ATP [20, 25–28]. As a result of the decreased overall ATP yield of sucrose dissimilation, less ATP is available for the energy-requiring production of biomass and glycerol, and therefore a larger fraction of the substrate is fermented to ethanol. In the resulting strain, the overall ATP yield was decreased from 4 to 3 ATP per mol of sucrose, which was reflected in a 33% decrease in biomass yield and an 11% increase in ethanol yield [21]. This increased ethanol yield on sucrose could be of relevance for industrial production of bioethanol from sucrose-rich sources, such as sugarcane and sugar beet, especially since the sucrose-containing feedstock can make up to 70% of the total process cost [29].

In theory, the ethanol yield in anaerobic *S. cerevisiae* cultures can be further improved by lowering the ATP yield of sucrose dissimilation in this yeast even more. Extracellular hydrolysis and subsequent uptake of the resulting monosaccharides by proton symport, instead of facilitated diffusion, would lower the yield from 4 to 2 ATP per mol of sucrose. In this study, we investigated whether the facilitated diffusion mechanism for hexose uptake could be replaced by hexose–proton symport in *S. cerevisiae*. To this end, two glucose– and two fructose–proton symporter variants were introduced in a strain background that is devoid of all native hexose transporter genes, and the physiology of the resulting strains when growing on the corresponding hexose was studied. Subsequently, we investigated whether these hexose–proton symporters could also be applied in a strain background in which sucrose is exclusively hydrolyzed extracellularly and uptake of the resulting monosaccharides is mediated via a proton symport mechanism.

## Results

### Expression of single hexose–proton symporters is sufficient to enable aerobic growth on glucose or fructose

**Fig. 1:**
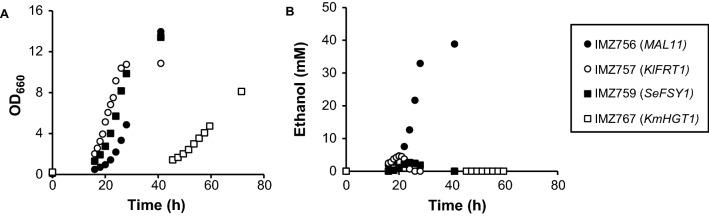
Growth (**A**) and ethanol production (**B**) of IMZ756 (*MAL11*), IMZ757 (*KlFRT1*), IMZ759 (*SeFSY1*) and IMZ767 (*KmHGT1*) in shake flasks with either SMD (IMZ756 and IMZ767) or SMF (IMZ757 and IMZ759) under aerobic conditions. Data shown represent one of two independent replicates

The first step towards the construction of *S. cerevisiae* strains that are dependent on proton-coupled hexose transport was the selection of suitable transporter candidates. *S. cerevisiae* possesses a native proton-coupled transporter with affinity for glucose (Mal11); however, this transporter also has affinity for maltose and sucrose [13, 30, 31]. Therefore, *FSY1* from *Saccharomyces eubayanus (SeFSY1)*, *FRT1* from *Kluyveromyces lactis (KlFRT1)* and KMXK_A02960 from *Kluyveromyces marxianus (KmHGT1)*, which have been described in previous literature as glucose– or fructose–proton symporters [32–36], were selected for heterologous expression in *S. cerevisiae*, in addition to *MAL11*. To be able to investigate strains in which the sole transport mechanism for hexose uptake is proton symport, without interference of transporters with a facilitated diffusion mechanism, the selected proton symporters were individually overexpressed in strain IMX2144, a derivative of IMX1812 [37], which is devoid of all native hexose transporters (*hxt*^*0*^). The resulting strains were grown aerobically in shake flasks with SM with 20 g L^−1^ glucose (SMD) or 20 g L^−1^ fructose (SMF) as the sole carbon source. No growth was observed for the empty vector-carrying strain IMZ796 after 10 days, which reflects the absence of any glucose or fructose transporters in *hxt*^*0*^-strain IMX2144. Overexpression of either *MAL11* or *KmHGT1* restored growth on glucose, and the corresponding strains had a specific growth rate (µ) of 0.19 ± 0.00 h^−1^ (*MAL11*) and 0.09 ± 0.00 h^−1^ (*KmHGT1*) on this carbon source, whereas no growth was observed for these strains in SMF after ten days. On the other hand, *SeFSY1* and *KlFRT1* restored growth on fructose (µ = 0.18 ± 0.00 h^−1^ and 0.23 ± 0.00 h^−1^, respectively), but not on glucose. Interestingly, only for strain IMZ756 (*MAL11*) aerobic fermentation was clearly observed, as apparent from the production of ethanol (up to 39 mM) in these cultures (Fig. 1). For IMZ757 (*KlFRT1*) and IMZ759 (*SeFSY1*), some ethanol was produced (up to 4.5 mM and 2.5 mM, respectively) in the early exponential phase, which was subsequently co-consumed with fructose.

### Laboratory evolution enables anaerobic growth on hexoses

The replacement of facilitated diffusion by proton symport of hexose uptake is expected to decrease the ATP yield of dissimilation by 1 ATP per glucose or fructose molecule due to the ATP requirement of proton extrusion. In this scenario, the resulting impact on the cellular energy metabolism and thereby strain physiology is more pronounced when the hexoses are fermented and only 2 ATP is produced per hexose, compared to the estimated 16 ATP per hexose for aerobic respiration [38, 39]. However, when investigated in anaerobic shake flasks (with SMD for *MAL11* and *KmHGT1*, SMF for *KlFRT1* and *SeFSY1*), only the *MAL11*-overexpressing strain IMZ756 grew on SMD, whereas no growth was observed for IMZ757 (*KlFRT1*), IMZ759 (SeFSY1) and IMZ767 (*KmHGT1*) after incubation for 10 days. Therefore, an evolutionary engineering strategy was applied to select for anaerobically growing mutants. To this end, all four strains were grown in sequential batch reactor (SBR) setups that were sparged with a mixture of air and nitrogen. Over the course of the evolution, the supply of air (and thus oxygen) was decreased stepwise until the strains exhibited growth under anaerobic conditions (Additional file 2). IMZ756 (*MAL11*) was also included in these evolution experiments to potentially improve its fermentation kinetics. Since this strain was already able to grow under anaerobic conditions, the switch from aerobic to anaerobic conditions was made without intermediate steps. After approximately 85 (*MAL11*), 106 (*KmHGT1*), 230 (*SeFSY1*) and 200 (*KlFRT1*) generations, the cultures were switched to fully anaerobic conditions, and after approximately 132 (*MAL11*), 30 (*KmHGT1*), 51 (*SeFSY1*) and 35 (*KlFRT1*) additional generations, single colonies were isolated from each reactor, resulting in IMS1058 (*MAL11*), IMS1059 (*KlFRT1*), IMS1060 (*SeFSY1*) and IMS1061 (*KmHGT1*).

### Replacement of hexose diffusion by proton-coupled hexose transport decreases the ATP yield of glucose and fructose fermentation

Subsequently, the four evolved strains were characterized in anaerobic chemostats, which were glucose-limited for IMS1058 (*MAL11*) and IMS1061 (*KmHGT1*) and fructose-limited for IMS1059 (*KlFRT1*) and IMS1060 (*SeFSY1*). As a reference, *HXT5,* encoding a hexose uniporter, was overexpressed in IMX2144 (*hxt*^*0*^) and the resulting strain was characterized in both glucose- and fructose-limited chemostats. From measurements of the CO_2_ concentration in the reactor off-gas during the preceding batch phase, the anaerobic maximum specific growth rates (µ_max_) were estimated (Table 1). Based on these growth rates, the flowrate of the ingoing medium was adjusted to obtain a dilution rate of 0.07 h^−1^ after the batch phase, so that all strains could be compared at the same growth rate. Compared to the *HXT5*-expressing reference strain, the biomass yield of the strains expressing hexose proton symporters was reduced by 44.0–47.6% (Table 1), which is close to the reduction of 50% that is expected when the energetic efficiency of dissimilation is also decreased by 50% (from 2 to 1 mol of ATP per mol of hexose) [26]. Consequently, since these strains were grown at identical growth rates, a twofold increase in the biomass-specific glucose consumption, ethanol production and CO_2_ production rates of the proton symporter-expressing strains were expected. Indeed, a fold-increase between 1.8 and 2.2 of these biomass-specific rates was observed for all proton-coupled strains (Table 1). Based on the ethanol yields that were measured for the *HXT5*-expressing reference strain, the ethanol yields of the proton symporter-expressing strains were predicted to be increased by 16.2% on glucose and 13.3% on fructose when hexose transport is completely proton coupled (Additional file 1: S1). In line with this prediction, compared to the reference strain, the ethanol yields of the strains carrying the glucose–proton symporters *MAL11* and *HGT1* were increased by 17.2% and 14.6%, respectively, while the ethanol yields of the strains carrying the fructose–proton symporters *FRT1* and *FSY1* were increased by 13.3% and 10.8%, respectively (Table 1). Differences in kinetic properties of the transporters were reflected in the varying residual sugar concentrations among the different strains, suggesting that *KmHGT1* exhibits the highest affinity for glucose (0.11 ± 0.00 g L^−1^) and *SeFSY1* the highest affinity for fructose (0.12 ± 0.02 g L^−1^, Table 1).

**Table 1:** Growth characteristics of anaerobically evolved *hxt*^*0*^-strains expressing proton symporters and a *hxt*^*0*^ strain expressing *HXT5* in either glucose- or fructose-limited anaerobic steady state chemostat cultures at a dilution rate of 0.07 h^−1^

Strain	IMZ763	IMS1058	IMS1061	IMZ763	IMS1059	IMS1060
Hexose transporter	*HXT5*	*MAL11*	*KmHGT1*	*HXT5*	*KlFRT1*	*SeFSY1*
Carbon source	Glucose	Glucose	Glucose	Fructose	Fructose	Fructose
µ_max_ (h^−1^) ^a^	0.22 ± 0.00	0.21 ± 0.01	0.13 ± 0.00	0.23 ± 0.00	0.16 ± 0.00	0.11 ± 0.00
Biomass yield (g_x_ g_s_^−1^)	0.084 ± 0.001	0.044 ± 0.000	0.047 ± 0.000	0.088 ± 0.003	0.047 ± 0.000	0.048 ± 0.001
Ethanol yield (mol mol_s_^−1^)	1.51 ± 0.01	1.77 ± 0.01	1.73 ± 0.00	1.58 ± 0.02	1.79 ± 0.01	1.75 ± 0.02
q_s_ (mmol g_x_^−1^ h^−1^)	-4.69 ± 0.06	-8.87 ± 0.15	-8.38 ± 0.16	-4.46 ± 0.13	-8.38 ± 0.02	-8.11 ± 0.36
q_ethanol_ (mmol g_x_^−1^ h^−1^)	7.08 ± 0.05	15.70 ± 0.13	14.53 ± 0.28	7.03 ± 0.10	14.98 ± 0.12	14.16 ± 0.50
q_glycerol_ (mmol g_x_^−1^ h^−1^)	0.59 ± 0.01	0.68 ± 0.00	0.59 ± 0.00	0.53 ± 0.00	0.58 ± 0.02	0.55 ± 0.03
q_CO2_ (mmol g_x_^−1^ h^−1^)	7.85 ± 0.06	15.91 ± 0.09	15.36 ± 0.33	7.32 ± 0.12	15.36 ± 0.05	14.88 ± 0.39
Residual sugar (g L^−1^)	0.16 ± 0.03	2.87 + 0.16	0.11 ± 0.00	0.93 ± 0.05	1.02 ± 0.02	0.12 ± 0.02
Carbon recovery (%) ^b^	96.4 ± 0.1	99.0 ± 1.1	98.4 ± 0.1	98.3 ± 1.4	99.8 ± 0.0	98.7 ± 1.2
Actual dilution rate (h^−1^)	0.071 ± 0.002	0.071 ± 0.001	0.071 ± 0.001	0.071 ± 0.004	0.071 ± 0.000	0.070 ± 0.001

^a^The maximum specific growth rate (µ_max_) was determined based on measurements of the CO_2_ concentration in the reactor off-gas during the preceding batch phase

Biomass-specific production and consumption rates are depicted as q_metabolite_

^b^The carbon recovery represents the percentage of the carbon entering the reactor system via the medium that could be traced back in biomass, products and residual substrate

The data represent average values and mean deviations obtained from duplicate experiments

### Development of a ‘sugar-negative’ *S. cerevisiae* strain devoid of all hexose transport and disaccharide transport and hydrolysis

**Fig. 2:**
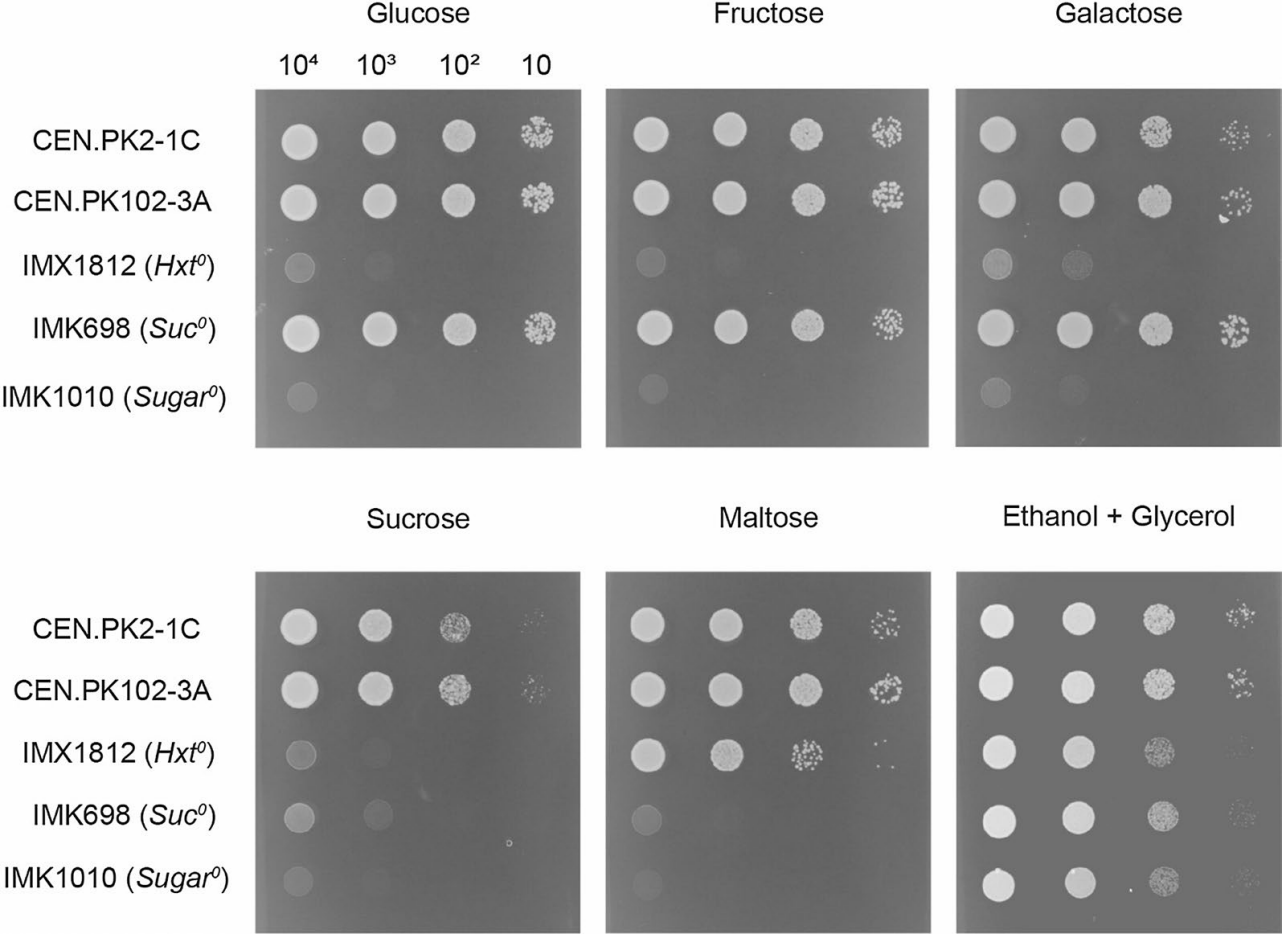
Growth of hexose transport-negative strain IMX1812, sucrose-negative strain IMK698 and sugar-negative strain IMK1010 and their ancestors (CEN.PK2-1C and CEN.PK102-3A) on SM with 20 g L^−1^ glucose, fructose, galactose, sucrose or maltose, or 2% (v/v) ethanol and 2% (v/v) glycerol. Pictures were taken after two days of incubation for the plates with glucose, fructose, maltose and sucrose, after three days of incubation for the plate with galactose and after eight days of incubation for the plate with ethanol and glycerol, all at 30 °C

The increased ethanol yields of strains expressing hexose–proton symporters (Table 1) could be of relevance to industrial ethanol production processes. In addition to (corn-derived) glucose, the disaccharide sucrose is widely used as sugar substrate for bioethanol production, especially in Brazilian sugarcane-based ethanol production plants [40]. Therefore, we aimed to investigate whether the combined expression of a glucose–proton symporter with a fructose–proton symporter also allowed for an increased ethanol yield on sucrose, similar to what was achieved on glucose and fructose (Table 1). For this strategy, sucrose should exclusively be hydrolyzed extracellularly, after which the resulting monosaccharides are taken up by proton-coupled transporters. To prevent the uptake and intracellular hydrolysis of sucrose, a platform strain was required that is devoid of all genes involved in sucrose transport and hydrolysis, on top of the absence of all native hexose transporter genes. Therefore, all hexose transporter genes were deleted in the previously constructed ‘sucrose-negative’ strain IMK698 (*Suc*^*0*^) [41] using a CRISPR–Cas9-based toolkit developed for the construction of a hexose transporter-deficient strain [37]. In IMK698, consumption of maltose and sucrose is completely abolished by deletion of all disaccharide transport and hydrolysis genes, whereas the strategy used by Wijsman et al. (2018) results in a strain that is unable to consume glucose, fructose and galactose (*Hxt*^*0*^). A combination of these two strategies resulted in the ‘sugar-negative’ strain IMK1010 (*Sugar*^*0*^), which contained deletions in 23 transporter genes and 9 hydrolysis genes. Growth of IMK1010 was compared to that of the previously constructed strains IMX1812 (*Hxt*^*0*^) and IMK698 (*Suc*^*0*^) and their parental strains CEN.PK2-1C and CEN.PK102-3A to test its ability to grow on any of the relevant sugar substrates (Fig. 2). Indeed, growth of IMK1010 was only observed on SM with ethanol and glycerol, whose transport and conversion are not dependent on any of the deleted genes, whereas growth on glucose, fructose, galactose, sucrose and maltose was absent.

### Functional replacement of native sucrose metabolism with exclusively extracellular hydrolysis and proton-coupled hexose uptake

Next, the (predominantly) extracellular invertase gene *SUC2* was integrated under control of the strong, constitutive *TDH3* promoter, after which a plasmid carrying both *KmHGT1* and *SeFSY1* was introduced. *KmHGT1* and *SeFSY1* were selected since they appeared to exhibit the highest affinity for their respective sugar (Table 1) and, in contrast to *MAL11*, were not expected to have any affinity for sucrose. The resulting strain, IMZ783 (*KmHGT1* and *SeFSY1*), was able to grow in SM with sucrose as the sole carbon source under aerobic conditions, but not under anaerobic conditions. Similar to the previous evolution with the single hexose–proton symporter-expressing strains, IMZ783 was evolved in duplicate SBRs on SM with 20 g L^−1^ sucrose, with a stepwise decreasing oxygen supply until growth under anaerobic conditions was achieved (Additional file 2). From both reactors, single colonies were isolated, resulting in IMS1214 and IMS1215. To investigate whether the engineering strategy indeed led to an increased ethanol yield, IMS1215 (which was isolated first), was grown in anaerobic, sucrose-limited chemostats and compared to IMZ785, an isogenic *SUC2*-expressing strain that overexpressed *HXT5* instead of *HGT1* and *FSY1*. In the preceding batch phase, the maximum specific growth rates were estimated from the CO_2_ concentration measurements in the off-gas. Since these estimated growth rates were 0.15 ± 0.00 h^−1^ for IMS1215 (*KmHGT1* and *SeFSY1*) and 0.28 ± 0.00 h^−1^ for IMZ785 (*HXT5*), the subsequent chemostats were operated at an identical dilution rate of 0.10 h^−1^. For both strains, no residual sucrose was detected in the culture supernatant, and it appeared to be completely hydrolyzed to glucose and fructose. As expected for a strain in which hexose diffusion has been replaced with proton-coupled transport, the biomass yield of IMS1215 (*KmHGT1* and *SeFSY1*) was decreased by 46.6% compared to the *HXT5*-expressing reference strain (Table 2), which is close to the theoretical 50% decrease [26]. This observation is in accordance with the biomass-specific rates of sucrose consumption, ethanol production and CO_2_ production, for which a fold-increase between 1.9 and 2.2 was observed. Consequently, the ethanol yield, which was predicted to increase by 16.2% (Additional file 1: S1), was increased from 1.51 to 1.76 mol ethanol mol hexose equivalent^−1^, which is a 16.6% increase.

**Table 2:** Growth characteristics of IMZ785 and IMS1215 in sucrose-limited anaerobic steady-state chemostat cultures at a dilution rate of 0.1 h^−1^

Strain	IMZ785	IMS1215
Relevant genotype	*HXT5*	*HGT1* + *FSY1*
Carbon source	Sucrose	Sucrose
µ_max_ (h^−1^) ^a^	0.28 ± 0.00	0.15 ± 0.00
Biomass yield (g_x_ g_hexose eq_^−1^)	0.088 ± 0.005	0.047 ± 0.000
Ethanol yield (mol mol_hexose eq_^−1^)	1.51 ± 0.00	1.76 ± 0.02
q_s_ (mmol g_x_^−1^ h^−1^)	6.41 ± 0.43	11.9 ± 0.23
q_ethanol_ (mmol g_x_^−1^ h^−1^)	9.71 ± 0.63	20.9 ± 0.19
q_glycerol_ (mmol g_x_^−1^ h^−1^)	0.86 ± 0.08	0.85 ± 0.02
q_CO2_ (mmol g_x_^−1^ h^−1^)	10.1 ± 0.76	20.2 ± 0.40
Residual sugar (g L^−1^)	0.40 ± 0.01 (glucose) 0.56 ± 0.07 (fructose)	0.70 ± 0.00 (glucose) 4.86 ± 0.10 (fructose)
Carbon recovery (%) ^b^	95.8 ± 0.10	97.7 ± 0.45
Actual dilution rate (h^−1^)	0.102 ± 0.001	0.102 ± 0.002

^a^The maximum specific growth rate (µ_max_) was determined based on measurements of the CO_2_ concentration in the reactor off-gas during the preceding batch phase

Biomass-specific production and consumption rates are depicted as q_metabolite_

^b^The carbon recovery represents the percentage of the carbon entering the reactor system via the medium that could be traced back in biomass, products and residual substrate

The data represent average values and mean deviations obtained from duplicate experiments

### Mutations in overexpressed transporter genes enabled growth under anaerobic conditions

**Fig. 3:**
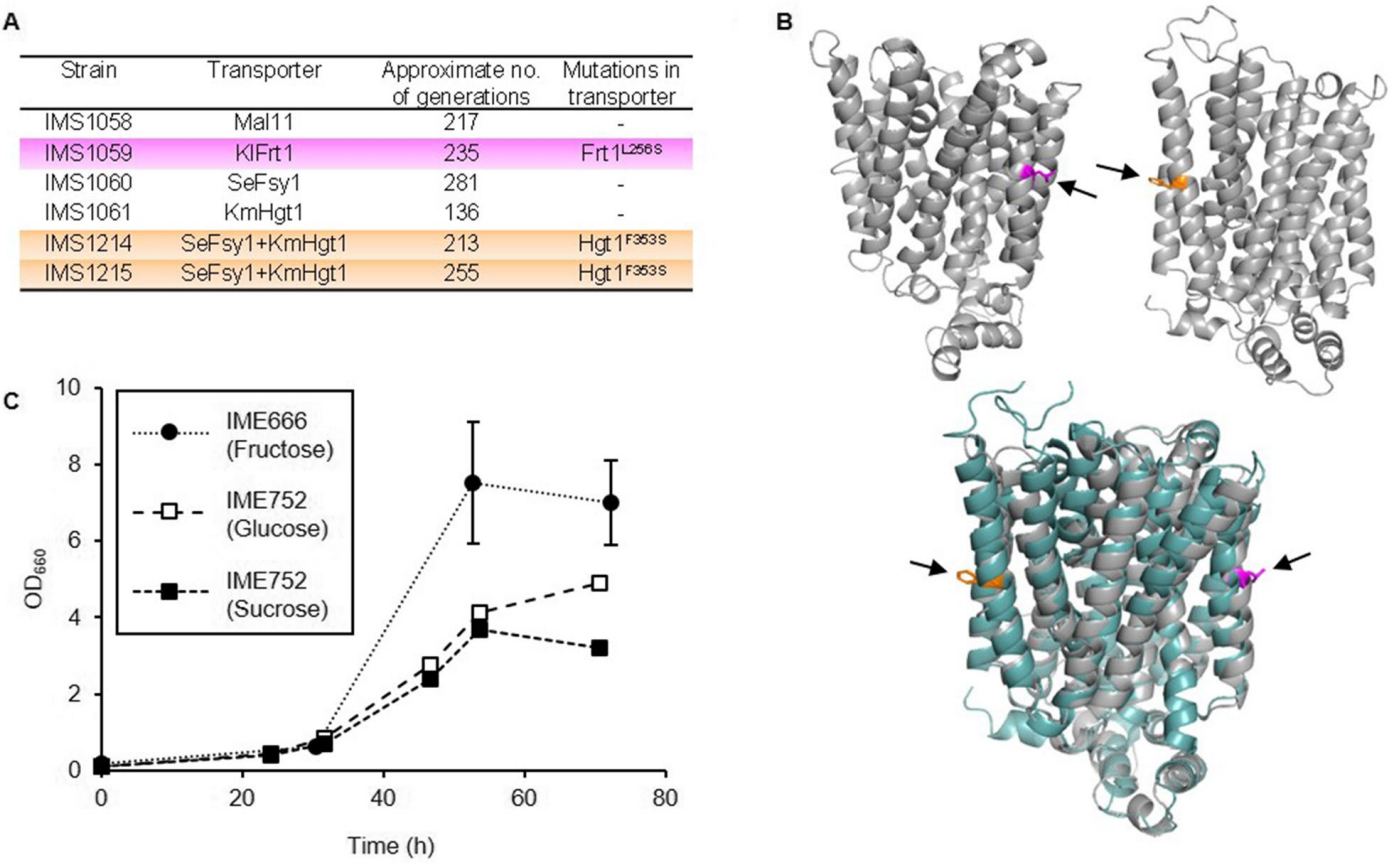
Evolution of strains expressing hexose–proton for growth under anaerobic conditions. **A** Mutations that arose in transporters during evolution of hexose–proton symporter-expressing strains under anaerobic conditions. **B** Structural models of KlFrt1 (top left) and KmHgt1 (top right) and both structures superimposed (bottom), highlighting the locations of amino acid residues that mutated during evolution in magenta and orange. **C** Growth of reverse engineered strains IME666 (*KlFRT1*^*T767C*^) in SM with 20 g L^−1^ fructose and IME752 (*KmHGT1*^*T1058C*^ and *SeFSY1*) in SM with 20 g L^−1^ sucrose or in SM with 20 g L^−1^ glucose in anaerobic shake flasks. Data represent average and mean deviation of two replicate experiments

The strains expressing heterologous transporters that were constructed in this study were only able to grow under anaerobic conditions after applying an evolutionary engineering approach. To investigate which mutations enabled anaerobic growth of the evolved strains, the genomes of single-colony isolates IMS1058 (*MAL11*), IMS1059 (*KlFRT1*), IMS1060 (*SeFSY1*), IMS1061 (*KmHGT1*), IMS1214 (*KmHGT1* and *SeFSY1*, first reactor) and IMS1215 (*KmHGT1* and *SeFSY1*, second reactor) were sequenced, along with their parental strains (IMX2144 and IMZ783) to identify single nucleotide polymorphisms (SNPs), insertions or deletions (indels) in coding sequences and copy number variations that occurred during the evolutions (Additional file 1: S3). Strikingly, whereas only few genomic mutations were identified in open reading frames, a SNP was found in (one of) the introduced transporter gene(s) in three of the strains: a thymine to cytosine substitution in position 767 of *KlFRT1* in IMS1059 (*KlFRT1*^*T767C*^), resulting in a leucine-to-serine substitution in amino acid position 256, and a thymine-to-cytosine substitution in position 1058 of *KmHGT1* in both IMS1214 and IMS1215 (*KmHGT1*^*T1058C*^), resulting in a phenylalanine-to-serine substitution in amino acid position 353 (Fig. 3A). The locations of these mutations were investigated through homology modeling of the corresponding protein structures, which predicted that both mutations occurred in the center of one of the membrane-facing transmembrane helices, at approximately the same height (Fig. 3B). To exclude that other genomic mutations were essential for anaerobic growth of the evolved strains, the plasmids containing the mutated transporter genes were isolated and reintroduced into an unevolved strain background, which was IMX2144 (*hxt*^*0*^) in case of the plasmid containing *KlFRT1*^*T767C*^ and IMX2719 (*sugar*^*0*^* SUC2*) in case of the plasmid containing *FSY1* and *KmHGT1*^*T1058C*^. Subsequently, the resulting *KlFRT1*^*T767C*^-expressing strain IME666 was grown in anaerobic shake flasks on SM with fructose as the sole carbon source, whereas anaerobic growth of the *FSY1-* and *KmHGT1*^*T1058C*^-expressing strain IME752 was tested in both SM with sucrose, in which the corresponding strain IMS1215 was evolved, and SM with glucose, which is the substrate of the mutated Hgt1 transporter. After a relatively short lag phase, both strains exhibited anaerobic growth, and cultures were fully grown within 54 h (Fig. 3), suggesting that the mutations in *KlFRT1* and in *KmHGT1* are largely responsible for the evolved phenotype of the corresponding strains.

## Discussion

In this study, we successfully replaced facilitated transport of hexoses by proton-coupled transport. In the resulting strains, the energetic difference between these two modes of transport was apparent by the yield of biomass and ethanol on sugar, which were up to 46.6% decreased and up to 17.2% increased, respectively, and close to the theoretical predicted values. Ethanol yield on carbohydrates is one of the main performance indicators for industrial bioethanol production. With an annual production volume of ~ 99 billion liters of ethanol in 2020 [40], even small improvements of the ethanol yield would have large economic benefits. Most of the annually produced bioethanol (~ 53%) originates from fermentation of corn starch-derived glucose in the USA [40], for which the strategy tested in this work, with strains that depend on a single glucose–proton symporter for their hexose uptake could be relevant. Another ~ 30% is produced from sugarcane-derived sucrose in Brazil [40], which prompted us to combine hexose–proton symport and extracellular hydrolysis in the sugar-negative strain. An additional advantage of this strategy is that the decrease in biomass yield also results in a lower yield of the by-product glycerol, which is formed to re-oxidize the ‘surplus’ of NADH that arises through biomass synthesis. It should be noted that the aforementioned beneficial effects on production of decreasing the ATP yield of dissimilation are growth rate dependent, since a larger fraction of sugar is dissimilated to meet growth rate-independent maintenance requirements at the lower growth rates [42]. Consequently, in industrial batch processes, lowering the ATP yield would be most beneficial in fermentation phases where high growth rates are observed, whereas the effect decreases towards the end of the fermentation, when growth ceases due to high concentrations of ethanol [43]. Additionally, for industrial implementation, engineered yeast cells should be able to cope with high maintenance energy requirements that arise due to such high ethanol concentrations and the presence of other inhibitors in the culture [43–46]. Therefore, in addition to other practical considerations for efficient use of such engineered strains in industrial fermentations [47], the expressed sugar transporters should allow for very high dissimilation rate for maintenance energy provision.

The strains in which heterologous transporters were introduced were unable to directly grow under anaerobic conditions. We hypothesize that an insufficient rate of ATP production might be underlying this absence of anaerobic growth in strains expressing *KmHGT1*, *KlFRT1* and *SeFSY1* for their sugar uptake. Although these strains exhibited reasonable growth rates (up to 0.23 h^−1^) under aerobic conditions (Fig. 1), the transition from respiration to fermentation, which in these strains corresponds to a transition from an estimated 15 ATP to 1 ATP per hexose equivalent, might have decreased the ATP production rate to below maintenance energy requirements. Unlike these strains, the *MAL11*-expressing strain IMZ756, without evolution exhibited aerobic fermentation (Fig. 1B), which suggests that (1) there is a smaller transition of the ATP yield between aerobic and anaerobic conditions for this strain, since under aerobic conditions, already part of the overall sugar dissimilation yields 1 ATP; and therefore (2) this strain should have exhibited a substantially higher sugar uptake rate than the other strains to achieve its similar aerobic growth rate. The latter could be confirmed by quantifying the biomass-specific sugar consumption rates of these strains in aerobic batch bioreactor cultures. Such a high sugar uptake rate might have enabled IMZ756 (*MAL11*) to grow directly under anaerobic conditions, without evolution. Moreover, since *MAL11* is the only endogenous transport gene tested, these observations might point out a difficulty in the functional expression of heterologous transporters in the anaerobic membrane of *S. cerevisiae*, which has a lower unsaturated fatty acid and ergosterol content than the membrane of aerobically grown *S. cerevisiae* [48–50]*.* Especially for transporters such as *KmHGT1* and *KlFRT1,* which originate from obligate aerobic yeasts [51, 52], their functional expression under anaerobic conditions could have been impacted by differences in membrane composition. Possibly, the evolution of the *KmHGT1-*, *KlFRT1-* and *SeFSY1*-expressing strains allowed for selection of mutant cells with an increased sugar uptake rate, thereby enabling a sufficient supply of free energy under anaerobic conditions. In case of IMS1059 (*KlFRT1*), IMS1214 and IMS1215 (both expressing *KmHGT1* and *SeFSY1*), mutations in transporter sequences probably underlie such an increased sugar uptake rate, since their reintroduction in an unevolved strain background allowed for immediate anaerobic growth. Both mutations led to the substitution of a hydrophobic residue (leucine or phenylalanine) in the center of one of the transmembrane helices to a (polar) serine residue (Fig. 3B). Further investigation of the molecular mechanism of these mutations could provide valuable information to facilitate the introduction of heterologous transporters. From whole genome sequencing data of IMS1060 (*SeFSY1*) and IMS1061 (*KmHGT1*) no clear leads could be found in SNPs and indels in coding regions or copy number variations that could be related to the evolved phenotype, and thus, the mutations underlying their evolved phenotype were not investigated via reverse engineering. The absence of mutations in *KmHGT1* in IMS1061 was especially striking since this gene was mutated in both IMS1214 and IMS1215 (*KmHGT1* and *SeFSY1* combined). Potentially, mutations outside of open reading frames, which were not analyzed in this research, led to changes in gene expression that allowed for anaerobic growth, which could be further elucidated in future work by transcriptome analysis.

Since transport by proton symporters is not only driven by the concentration gradient of the solute, but also by the proton motive force, these transporters allow for the intracellular accumulation of sugars, and therefore theoretically allow for growth at lower extracellular sugar concentrations. This property can be advantageous in industrial fermentations (not necessarily bioethanol), as it decreases the likelihood of culture contamination by competing microorganisms that require higher extracellular sugar concentrations [53–55]. Another advantage for sucrose-grown cultures is that expression of proton-coupled hexose transporters could prevent the accumulation of fructose at the end of cultivation processes, which often occurs due to preferred uptake of glucose over fructose by wild-type *S. cerevisiae* [56–58]. The residual glucose and fructose concentrations that were measured in this study for strains expressing proton symporters were not (substantially) lower than previously measured for reference strain CEN.PK113-7D [59], indicating that the kinetics of glucose and fructose transport were not improved by expression of the transporters that were investigated in this study. High-throughput screening of heterologous proton symporters may lead to the identification of transporters with better kinetics when expressed in *S. cerevisiae*. Alternatively, another evolutionary engineering approach could be applied, in which prolonged cultivation of the strains constructed in this study in continuous cultures under substrate limitation may lead to selection of mutants with improved performance. This approach has been successfully applied to improve the uptake of the disaccharides sucrose [21] and maltose [60].

Although this work was focused on increasing the ethanol yield on sugar, engineering strategies should not compromise other performance indicators such as productivity and titer, even under the harsh conditions that cells experience in industrial fermentations. However, the maximum specific growth rates of the strains developed in this study in anaerobic cultures (0.11–0.21 h^−1^) were still considerably lower than that of a wild-type strain (0.42 h^−1^ [61]). Prolonged evolution in a sequential batch reactor setup may be a suitable strategy to further select for mutants with faster sugar uptake rates and, consequently, higher ATP production rates. Since both the growth rate and the affinity for sugars are important targets for optimization, a laboratory evolution setup should ideally select for both characteristics simultaneously. Desired mutants could therefore be selected in an accelerostat setup: a continuous culture in which the dilution rate is feed-back controlled based on continuous online measurements (for example the CO_2_ concentration in the reactor off gas) [62, 63].

To the best of our knowledge, the platform strain IMK1010 (*sugar*^*0*^) constructed in this study is the first reported *S. cerevisiae* strain that is completely devoid of all sugar transporters and disaccharide hydrolases. This strain can form a valuable basis for thorough investigation of the function of individual sugar transporters, especially for those with a broad substrate range. The absence of any other sugar transport activity allows for complementation studies and for the conductance of transport assays without ‘background signal’. In addition, this strain can be used in engineering strategies aimed at substrate liberation and uptake and associated (directed) evolution strategies. For instance, IMK1010 could be used to combine extracellular maltose hydrolysis and glucose–proton symport to alter the efficiency of maltose dissimilation, which could be of relevance in the baking and beer brewing industry [64].

## Conclusions

In this study, we showed that the replacement of the hexose transporters with a facilitated diffusion mechanism in *S. cerevisiae* by glucose–proton symporters (*MAL11* or *KmHGT1*) or fructose–proton symporters (*KlFRT1* or *SeFSY1*) results in a substantial (~ 17%) increase in the ethanol yield on glucose and fructose, respectively, at the cost of biomass synthesis. Although the expression of heterologous transporters could initially only restore aerobic growth on the corresponding hexose in an *hxt*^*0*^ strain background, anaerobically growing mutants for each of the tested transporters were obtained via evolutionary engineering. By combining the expression of an extracellular sucrose hydrolase, the glucose–proton symporter *KmHGT1* and fructose–proton symporter *SeFSY1* in a novel platform strain, devoid of all native hexose transporters and disaccharide transporters and hydrolases, a similar increase in ethanol yield and decrease in biomass yield was obtained in anaerobic cultures grown on sucrose. These results show the potential of engineering the energy coupling mechanism of sugar transport in strains used for industrial bioethanol production. Moreover, the novel ‘sugar-negative’ platform strain constructed in this study could be valuable for the development of yeast cell factories for other bioproducts or investigation of endogenous and heterologous sugar transporters in future studies, especially those focusing on transport proteins that mediate the uptake of multiple different types of sugars.

## Materials and methods

### Strains and maintenance

All *Saccharomyces cerevisiae* strains used in this study are derived from the CEN.PK lineage [65]. For long-term storage, glycerol was added to cells that were grown in synthetic medium (see “Mediaandcultivation” section) until late exponential phase, to obtain a final concentration of 30% (v/v) glycerol, after which 1 mL aliquots were stored at -80 °C. Plasmids were propagated in *Escherichia coli* XL1-Blue cells (Agilent, Santa Clara, CA, USA), which were also stored at − 80 °C after addition of glycerol to a final concentration of 25% (v/v) to overnight cultures.

### Molecular biology techniques

Plasmids were isolated from *E. coli* cells using GeneJET Plasmid Miniprep Kit (Thermo Fisher Scientific). *S. cerevisiae* genomic DNA was isolated as previously described, using 0.2 M LiAc and 1% SDS to lyse the cells [66]. Genomic DNA from yeast species other than *S. cerevisiae* was isolated using the Yeastar Genomic DNA Isolation Kit (Baseclear, Leiden, The Netherlands). Amplification of DNA fragments for the purpose of cloning was performed using Phusion High Fidelity DNA polymerase (Thermo Fisher Scientific, Waltham, MA, USA), and for diagnostic PCRs, DreamTaq PCR Mastermix (Thermo Fisher Scientific) was used, both according to the manufacturer’s protocol. Oligonucleotide primers were purchased from Sigma Aldrich (Saint Louis, MO, USA) either PAGE purified (for cloning purposes), or desalted (for diagnostic purposes) (Additional File 1, S2). DNA fragments were separated and visualized on 1% agarose TAE gel by 30 min electrophoresis at 100 V. DNA fragments excised from this gel were isolated using the Zymoclean Gel DNA recovery Kit (Baseclear), whereas the GeneJET PCR Purification Kit (Thermo Fisher Scientific) was used for isolation of DNA fragments directly from the PCR reaction mixture. Gibson assembly of plasmids was performed using the NEBuilder HiFi DNA Assembly Master Mix (New England Biolabs, Ipswich, MA, USA) according to the manufacturer’s instructions, after which 1 µL of this mixture was introduced into XL1-Blue chemically competent *E. coli* cells (Agilent) for plasmid propagation, according to the manufacturer’s protocol. Transformation of *S. cerevisiae* strains was performed using LiAC/ssDNA/PEG, as previously described [67]. After transformation, single colonies were re-streaked three consecutive times to ensure an isogenic single cell line. Genomic deletions and integrations were confirmed by performing diagnostic PCR using genomic DNA of transformants as template. gRNA plasmids were removed from strains as described previously [68]. Plasmids were isolated from yeast strains using the Zymoprep Yeast Plasmid Miniprep II kit (Baseclear).

### Plasmid construction

All plasmids used in this study are listed in Table 3.

**Table 3:** Plasmids used in this study

Plasmid name	Relevant genotype	Source
p426TEF	2 μm ampR *URA3 pTEF1-tCYC1*	[69]
pUDE432	2 μm ampR *URA3 pTEF1-MAL11-tCYC1*	[41]
pUDE897	2 μm ampR *URA3 pTEF1-KlFRT1-tCYC1*	This study
pUDE898	2 μm ampR *URA3 pTEF1-SeFSY1-tCYC1*	This study
pUDE914	2 μm ampR *URA3 pTEF1-HXT5-tCYC1*	This study
pUDE920	2 μm ampR *URA3 pTEF1-KmHGT1-tCYC1*	This study
pUDE206	2 μm ampR *natNT1 pTPI1-I-SceI-tTEF1*	[70]
pUDE1024	2 μm ampR *URA3 pTPI1-SeFSY1-tTEF1*	This study
pUDE1028	2 μm ampR *URA3 pTEF1-KmHGT1-tCYC1 pTPI1-SeFSY1-tTEF1*	This study
p414-TEF1p-Cas9-CYC1t	*CEN6/ARS4* ampR *TRP1 pTEF1-cas9-tCYC1*	[71]
pUG-natNT2	ampR *loxP-natNT2-loxP*	[72]
pROS12	2 μm ampR *hphNT1* gRNA-*CAN1*.Y gRNA-*ADE2*.Y	[68]
pUDR313	2 μm ampR *hphN* gRNA-*HIS3* gRNA-*mTurquoise2*	This study
pROS10	2 μm ampR *URA3* gRNA-*CAN1*.Y gRNA-*ADE2*.Y	[68]
pUDR766	2 μm ampR *URA3* gRNA-*kanMX* gRNA-*kanMX*	This study
pUDR211	2 μm ampR *KlLEU2* gRNA-*HXT8* gRNA-*HXT14*	[37]
pUDR295	2 μm ampR *HIS3* gRNA-*GAL2* gRNA-*HXT4-1–5*;*HXT3-6–7*	[37]
pRS416	*CEN6/ARS4* ampR *URA3*	[73]
pUDR214	2 μm ampR *URA3* gRNA-*HXT13-15–16* gRNA-*HXT2*	[37]
pUDR220	2 μm ampR *KlLEU2* gRNA-*HXT10* gRNA-*HXT9-11–12*	[37]
pUDR418	2 μm ampR *URA3* gRNA-*STL1* gRNA-*STL1*	[37]
pUDE262	2 μm ampR *URA3 pTDH3-LmSPase-tADH1*	[41]
pUDE1103	2 μm ampR *URA3 pTDH3-SUC2-tADH1*	This study
pUDR119	2 μm ampR *amdS* gRNA-*SGA1*	[74]
pUDE1089	2 μm ampR *URA3 pTEF1-KlFRT1*^T767C^ *-tCYC1*	This study
pUDE1221	2 μm ampR *URA3 pTEF1-KmHGT1*^T1058C^*-tCYC1 pTPI1-SeFSY1-tTEF1*	This study

The prefixes ‘*Se*’, ‘*Kl*’ and ‘*Km*’ indicate genes originating from *Saccharomyces eubayanus*, *Kluyveromyces lactis* and *Kluyveromyces marxianus*, respectively

For construction of plasmids pUDE897, pUDE898, pUDE914 and pUDE920 via Gibson assembly, the backbone of pUDE432 was amplified using primers 7812 and 7999. *KlFRT1 (*GenBank AJ315952.1*)* was amplified from *Kluyveromyces lactis* CBS 2359 genomic DNA using primers 15,985 and 15,986, and subsequently assembled with the pUDE432 backbone, resulting in plasmid pUDE897. *SeFSY1 (*GenBank HE858449.1*)* was amplified from *Saccharomyces eubayanus* CBS 12,357 genomic DNA using primers 15,987 and 15,988, and subsequently assembled with the pUDE432 backbone, resulting in plasmid pUDE898. *HXT5* was amplified from CEN.PK113-7D genomic DNA using primers 16,387 and 16,388, and subsequently assembled with the pUDE432 backbone, resulting in plasmid pUDE914. *KmHGT1 (KMXK_A02960* in Varela et al. 2019*)* was amplified from *Kluyveromyces marxianus* CBS 6556 genomic DNA using primers 16,508 and 16,509, and subsequently assembled with the pUDE432 backbone, resulting in plasmid pUDE920.

Plasmids pUDE1024 and pUDE1025 were constructed by Gibson assembly of the pUDE206 backbone, amplified using primers 6486 and 9719, with *SeFSY1*, amplified from pUDE898 using primers 17,347 and 17,348, or with *KlFRT1*, amplified from pUDE897 using primers 17,349 and 17,350, respectively. For construction of plasmids pUDE1026 and pUDE1027, pUDE432 was linearized by PCR using primers 10,307 and 10,308, and assembled with the *SeFSY1* expression cassette, amplified from pUDE1024 using primers 10,305 and 10,306, or with the *KlFRT1* expression cassette, amplified from pUDE1025 using primers 10,305 and 10,306, respectively. Similarly, for construction of plasmids pUDE1028 and pUDE1029, pUDE920 was linearized using primers 10,307 and 10,308, and assembled with the *SeFSY1* expression cassette, amplified from pUDE1024 using primers 10,305 and 10,306, or with the *KlFRT1* expression cassette, amplified from pUDE1025 using primers 10,305 and 10,306, respectively.

pUDR313 and pUDR766 were constructed as described previously [68]. The backbone of pROS12 was amplified using primer 6005 and the 2-µm fragment was amplified from pROS12 using primers 10,519 and 11,826. Gibson assembly of the two resulting DNA fragments resulted in pUDR313. The backbone of pROS10 was amplified using primer 6005 and the 2-µm fragment was amplified from pROS10 using primer 12,743, which contains the gRNA sequence for targeting *KanMX*. Gibson assembly of the two resulting DNA fragments resulted in pUDR766.

For construction of plasmid pUDE1103, the backbone of pUDE262 was amplified using primers 17,586 and 17,587, and *SUC2* was amplified from CEN.PK113-7D genomic DNA using primers 17,584 and 17,585. Gibson assembly of the pUDE262 backbone and *SUC2* insert resulted in pUDE1103.

### Strain construction

All strains used in this study are listed in Table 4.

**Table 4:** Strains used in this study

Strain name	Relevant genotype	Source
CEN.PK113-7D	*MATa URA3 HIS3 LEU2 TRP1 MAL2-8c SUC2*	
*Kluyveromyces lactis* CBS 2359	Wild type	CBS-KNAW
*Saccharomyces eubayanus* CBS 12,357	Wild type	CBS-KNAW
*Kluyveromyces marxianus* CBS 6556	Wild type	CBS-KNAW
IMX1812	*MATa ura3-52 trp1-1 leu2-3,112 his3Δ can1::Spcas9-natNT2 gal2Δ hxt4-1-5Δ hxt3-6–7::ars4 hxt8Δ hxt14Δ hxt2Δ hxt9Δ hxt10Δ hxt11Δ hxt12Δ hxt13Δ hxt15Δ hxt16Δ mph2Δ mph3Δ mal11Δ stl1Δ*	[37]
IMX2115	*MATa ura3-52 trp1-1 leu2-3,112 ****HIS3**** can1::Spcas9-natNT2 gal2Δ hxt4-1-5Δ hxt3-6–7::ars4 hxt8Δ hxt14Δ hxt2Δ hxt9Δ hxt10Δ hxt11Δ hxt12Δ hxt13Δ hxt15Δ hxt16Δ mph2Δ mph3Δ mal11Δ stl1Δ*	This study
IMX2125	*MATa ura3-52 trp1-1 ****LEU2**** HIS3 can1Δ::Spcas9-natNT2 gal2Δ hxt4-1-5Δ hxt3-6–7::ars4 hxt8Δ hxt14Δ hxt2Δ hxt9Δ hxt10Δ hxt11Δ hxt12Δ hxt13Δ hxt15Δ hxt16Δ mph2Δ mph3Δ mal11Δ stl1Δ*	This study
IMX2144	*MATa ura3-52 ****TRP1**** LEU2 HIS3 can1::Spcas9-natNT2 gal2Δ hxt4-1-5Δ hxt3-6–7::ars4 hxt8Δ hxt14Δ hxt2Δ hxt9Δ hxt10Δ hxt11Δ hxt12Δ hxt13Δ hxt15Δ hxt16Δ mph2Δ mph3Δ mal11Δ stl1Δ*	This study
IMZ756	IMX2144 + pUDE432 (*URA3 MAL11*)	This study
IMZ757	IMX2144 + pUDE897 (*URA3 KlFRT1*)	This study
IMZ759	IMX2144 + pUDE898 (*URA3 SeFSY1*)	This study
IMZ763	IMX2144 + pUDE914 (*URA3 HXT5*)	This study
IMZ767	IMX2144 + pUDE920 (*URA3 KmHGT1*)	This study
IMZ796	IMX2144 + p426GPD (*URA3*)	This study
IMS1058	Single-colony isolate of anaerobically evolved IMZ756	This study
IMS1059	Single-colony isolate of anaerobically evolved IMZ757	This study
IMS1060	Single-colony isolate of anaerobically evolved IMZ759	This study
IMS1061	Single-colony isolate of anaerobically evolved IMZ767	This study
IMK698	*MATa ura3-52 leu2-112 MAL2-8C mal11-mal12::loxP mal21-mal22::loxP mal31-mal32::loxP mph2/3::loxP mph2/3::loxP-hphNT1-loxP suc2::loxP-kanMX-loxP ima1Δ ima2Δ ima3Δ ima4Δ ima5Δ*	[41]
IMX2572	*MATa ura3-52 leu2-112 MAL2-8C mal11-mal12::loxP mal21-mal22::loxP mal31-mal32::loxP mph2/3::loxP mph2/3::loxP-hphNT1-loxP suc2::loxP-kanMX-loxP ima1Δ ima2Δ ima3Δ ima4Δ ima5Δ ****can1::cas9-natNT2***	This study
IMK980	*MATa ura3-52 leu2-112 MAL2-8C mal11-mal12::loxP mal21-mal22::loxP mal31-mal32::loxP mph2/3::loxP mph2/3::loxP-hphNT1-loxP ****suc2::loxP**** ima1Δ ima2Δ ima3Δ ima4Δ ima5Δ can1::cas9-natNT2*	This study
IMK983	*MATa ura3-52 leu2-112 ****his3Δ**** MAL2-8C mal11-mal12::loxP mal21-mal22::loxP mal31-mal32::loxP mph2/3::loxP mph2/3::loxP-hphNT1-loxP suc2::loxP ima1Δ ima2Δ ima3Δ ima4Δ ima5Δ can1::cas9-natNT2*	This study
IMK985	*MATa ura3-52 leu2-112 his3Δ MAL2-8C mal11-mal12::loxP mal21-mal22::loxP mal31-mal32::loxP mph2/3::loxP mph2/3::loxP-hphNT1-loxP suc2::loxP ima1Δ ima2Δ ima3Δ ima4Δ ima5Δ can1::cas9-natNT2 ****hxt8Δ hxt14Δ gal2Δ hxt4Δ hxt1Δ hxt5Δ hxt3Δ hxt6Δ hxt7Δ***	This study
IMK990	*MATa ura3-52 leu2-112 his3Δ MAL2-8C mal11-mal12::loxP mal21-mal22::loxP mal31-mal32::loxP mph2/3::loxP mph2/3::loxP-hphNT1-loxP suc2::loxP ima1Δ ima2Δ ima3Δ ima4Δ ima5Δ can1::cas9-natNT2 hxt8Δ hxt14Δ gal2Δ hxt4Δ hxt1Δ hxt5Δ hxt3Δ hxt6Δ hxt7Δ ****hxt13Δ hxt15Δ hxt16Δ hxt2Δ***	This study
IMK992	*MATa ura3-52 leu2-112 his3Δ MAL2-8C mal11-mal12::loxP mal21-mal22::loxP mal31-mal32::loxP mph2/3::loxP mph2/3::loxP-hphNT1-loxP suc2::loxP ima1Δ ima2Δ ima3Δ ima4Δ ima5Δ can1::cas9-natNT2 hxt8Δ hxt14Δ gal2Δ hxt4Δ hxt1Δ hxt5Δ hxt3Δ hxt6Δ hxt7Δ hxt13Δ hxt15Δ hxt16Δ hxt2Δ ****hxt10Δ hxt9Δ hxt11Δ hxt12Δ***	This study
IMK1003	*MATa ura3-52 leu2-112 his3Δ MAL2-8C mal11-mal12::loxP mal21-mal22::loxP mal31-mal32::loxP mph2/3::loxP mph2/3::loxP-hphNT1-loxP suc2::loxP ima1Δ ima2Δ ima3Δ ima4Δ ima5Δ can1::cas9-natNT2 hxt8Δ hxt14Δ gal2Δ hxt4Δ hxt1Δ hxt5Δ hxt3Δ hxt6Δ hxt7Δ hxt13Δ hxt15Δ hxt16Δ hxt2Δ hxt10Δ hxt9Δ hxt11Δ hxt12Δ ****stl1Δ***	This study
IMK1008	*MATa ura3-52 ****LEU2**** his3Δ MAL2-8C mal11-mal12::loxP mal21-mal22::loxP mal31-mal32::loxP mph2/3::loxP mph2/3::loxP-hphNT1-loxP suc2::loxP ima1Δ ima2Δ ima3Δ ima4Δ ima5Δ can1::cas9-natNT2 hxt8Δ hxt14Δ gal2Δ hxt4Δ hxt1Δ hxt5Δ hxt3Δ hxt6Δ hxt7Δ hxt13Δ hxt15Δ hxt16Δ hxt2Δ hxt10Δ hxt9Δ hxt11Δ hxt12Δ stl1Δ*	This study
IMK1010	*MATa ura3-52 LEU2 ****HIS3**** MAL2-8C mal11-mal12::loxP mal21-mal22::loxP mal31-mal32::loxP mph2/3::loxP mph2/3::loxP-hphNT1-loxP suc2::loxP ima1Δ ima2Δ ima3Δ ima4Δ ima5Δ can1::cas9-natNT2 hxt8Δ hxt14Δ gal2Δ hxt4Δ hxt1Δ hxt5Δ hxt3Δ hxt6Δ hxt7Δ hxt13Δ hxt15Δ hxt16Δ hxt2Δ hxt10Δ hxt9Δ hxt11Δ hxt12Δ stl1Δ*	This study
IMX2719	*MATa ura3-52 LEU2 HIS3 MAL2-8C mal11-mal12::loxP mal21-mal22::loxP mal31-mal32::loxP mph2/3::loxP mph2/3::loxP-hphNT1-loxP suc2::loxP ima1Δ ima2Δ ima3Δ ima4Δ ima5Δ can1::cas9-natNT2 hxt8Δ hxt14Δ gal2Δ hxt4Δ hxt1Δ hxt5Δ hxt3Δ hxt6Δ hxt7Δ hxt13Δ hxt15Δ hxt16Δ hxt2Δ hxt10Δ hxt9Δ hxt11Δ hxt12Δ stl1Δ ****sga1::SUC2***	This study
IMZ783	IMX2719 + pUDE1028 (*URA3 KmHGT1 SeFSY1*)	This study
IMZ785	IMX2719 + pUDE914 (*URA3 HXT5*)	This study
IMS1214	Single-colony isolate of anaerobically evolved IMZ783	This study
IMS1215	Single-colony isolate of anaerobically evolved IMZ783	This study
IME666	IMX2144 + pUDE1089 (*URA3 KlFRT1*^T767C^)	This study
IME752	IMX2719 + pUDE1221 (*URA3 KmHGT1*^T1058C^ *SeFSY1)*	This study

Unless specified otherwise, these concern *S. cerevisiae* strains

*Saccharomyces eubayanus* strain CBS 12,357 (CRUB 1568; PYCC 6148), *Kluyveromyces lactis* strain CBS 2359 (ATCC 8585; CCRC 21,716; DBVPG 6725; DBVPG 6731; DBVPG 6833; DSM 70,799; IFO 1267; NRRL Y-1140; VKPM Y 1174) and *Kluyveromyces marxianus* strain CBS 6556 (ATCC 26,548; NCYC 2597; 359 NRRL Y-7571) were obtained from the Westerdijk Fungal Biodiversity 360 Institute (Utrecht, The Netherlands).

To restore histidine prototrophy in IMX1812, *HIS3* was amplified from CEN.PK113-7D genomic DNA using primers 1738 and 3755 and introduced into IMX1812, resulting in IMX2115. To restore leucine prototrophy in IMX2115, *LEU2* was amplified from CEN.PK113-7D genomic DNA using primers 1742 and 1743 and introduced into IMX2115, resulting in IMX2125. The tryptophan auxotrophy in this strain (*trp1-1*) is the result of a single point mutation in the *TRP1* gene [75]. By plating on medium without tryptophan, colonies were obtained that spontaneously reverted this mutation, resulting in strain IMX2144. Introduction of plasmids pUDE432, pUDE897, pUDE898, pUDE914, pUDE920 and p426TEF into IMX2144 resulted in strains IMZ756, IMZ757, IMZ759, IMZ763, IMZ767 and IMZ796, respectively.

IMZ756, IMZ757, IMZ759 and IMZ767 were each evolved in bioreactors to select for growth under anaerobic conditions (see below). From each individual bioreactor, single colonies were isolated by plating on SM with glucose (IMZ756 and IMZ767) or SM with fructose (IMZ757 and IMZ759) and re-streaking three consecutive times on the same medium, resulting in IMS1058, IMS1059, IMS1060 and IMS1061, respectively.

To facilitate genetic engineering of IMK698, *cas9* was integrated into its genome. To this end, *cas9* was amplified from p414-*TEF1*p-*cas9*-*CYC1*t using primers 2873 and 4653, and the *natNT2* marker was amplified from pUG-natNT2 using primers 3093 and 5542. Both DNA fragments were introduced into IMK698, resulting in IMX2572. Prior deletions in this strain were performed using the Cre-*loxP* system [76], but for the last deletion (*suc2*), the marker (*kanMX*) had not been recycled. To stimulate loop out of the *loxP*-flanked *kanMX* expression cassette, IMX2572 was transformed with pUDR766, resulting in IMK980. Further Cas9-assisted gene deletions were performed as previously described [68]. In IMK980, *HIS3* was deleted by introducing pUDR313, along with a double stranded DNA repair fragment obtained by annealing primers 10,521 and 10,522, resulting in strain IMK983. For deletion of all hexose transporters, the previously designed Cas9-assisted toolkit was used [37]. pUDR211 and pUDR295 were introduced into IMK983 along with five double stranded DNA repair fragments, obtained by annealing primers 9532 and 9533, 9551 and 9552, 9563 and 9564, 9522 and 9523 and by amplification from plasmid pRS416 with primers 9525 and 9526. The resulting strain was named IMK985. pUDR214 was introduced into IMK985, along with double stranded DNA repair fragments, obtained by annealing primers 9547 with 9548, 9555 with 9556, and 9528 with 9529, resulting in strain IMK990. pUDR220 was introduced into IMK990, along with double stranded DNA repair fragments, obtained by annealing primers 9538 with 9539, 9535 with 9536 and 9542 with 9543, resulting in strain IMK992. Finally, pUDR418 was introduced into IMK992, along with a double stranded DNA repair fragment, obtained by annealing primers 13,617 and 13,618, resulting in strain IMK1003. To regain leucine prototrophy, *LEU2* was amplified from CEN.PK113-7D genomic DNA using primers 1742 and 1743 and introduced into IMK1003, resulting in strain IMK1008. To regain histidine prototrophy, *HIS3* was amplified from CEN.PK113-7D genomic DNA using primers 1738 and 3755 and introduced into IMK1008, resulting in strain IMK1010.

A *SUC2* integration cassette was amplified from pUDE1103 using primers 9355 and 9356 and introduced into IMK1010, along with plasmid pUDR119, resulting in IMX2719. Introduction of plasmids pUDE1028 and pUDE914 into IMX2719 resulted in strains IMZ783 and IMZ785, respectively.

Plasmid pUDE1089 was isolated from IMS1059 and introduced into IMX2144, resulting in IME666. Similarly, plasmid pUDE1221 was isolated from IMS1215 and introduced into IMX2719, resulting in IME752.

### Media and cultivation

*E. coli* cultures were grown at 37 °C in LB medium, supplemented with 100 μg mL^−1^ ampicillin for selection and maintenance of plasmids. Yeast strains were grown on synthetic medium (SM), which was heat sterilized for 20 min at 121 °C, after which a filter-sterilized vitamin solution was added [77]. Depending on the transporters expressed, the medium was supplemented with either 20 g L^−1^ glucose and/or 20 g L^−1^ fructose (both added from 500 g L^−1^ stock solutions that were heat sterilized for 20 min at 110 °C), 20 g L^−1^ sucrose (added from a filter-sterilized 500 g L^−1^ solution) or 2% (v/v) ethanol in combination with 2% (v/v) glycerol (added from a 99% (v/v) solution that was heat sterilized for 20 min at 121 °C). For complementation of auxotrophic requirements, either 150 mg L^−1^ uracil, 100 mg L^−1^ histidine, 500 mg L^−1^ leucine and/or 75 mg L^−1^ tryptophan was added [78]. Medium for anaerobic cultivations was additionally supplemented with 10 mg L^−1^ ergosterol and 420 mg L^−1^ Tween 80, which were added in from a concentrated solution (800x) in absolute ethanol [79]. For preparation of solid medium plates, 2% (w/v) agar was added to the media prior to heat sterilization. Medium used in bioreactor cultivations was additionally supplemented with 0.2 g L^−1^ Antifoam C (Sigma Aldrich). Aerobic shake flask cultures were grown in 500 mL round bottom flasks with 100 mL medium, which were incubated in a New Brunswick Scientific Innova 44 Incubator Shaker (Eppendorf, Nijmegen, the Netherlands) at 200 rpm at 30 °C. Anaerobic shake flask cultures were grown at 30 °C in a Bactron anaerobic chamber with an atmosphere of 5% (v/v) H_2_, 6% (v/v) CO_2_ and 89% (v/v) N_2_, on a IKA KS 260 basic shaker (IKA-Werke GmbH & Co, Staufen, Germany) at 200 rpm, using 50 mL Erlenmeyer flasks containing 30 mL medium. For preparation of spot plates, strains were pre-grown on YP with 2% ethanol and 2% glycerol. Exponentially growing cells were collected by centrifugation (3000 g, 5 min at 4 °C), washed with sterile water and resuspended to a concentration of 10^7^ cells mL^−1^, determined using a Z2 Coulter Counter (Beckman Coulter, Woerden, the Netherlands) using a 50 μm aperture. Subsequently, dilutions were made of 10^6^, 10^5^, 10^4^ and 10^2^ cells mL^−1^, of which 10 μL was spotted on solid medium plates. Glucose-, fructose-, sucrose- and maltose-containing plates were incubated for 2 days, galactose-containing plates for three days and ethanol and glycerol-containing plates for eight days, all at 30 °C.

Laboratory evolution of IMZ756, IMZ757, IMZ759 and IMZ767 was conducted in Minifors 2 bioreactors (INFORS HT, Velp, the Netherlands) with a working volume of 100 mL in a sequential batch reactor (SBR) setup. IMZ756 and IMZ767 were evolved in SM with 20 g L^−1^ glucose, whereas IMZ757 and IMZ759 were evolved in SM with 20 g L^−1^ fructose. The cultures were stirred at 800 rpm and the temperature was kept constant at 30 °C. The pH was maintained at 5.0 by automated addition of a 2.0 M KOH solution. Cultures were sparged with 50 mL min^−1^ of a gas mixture of air and nitrogen. Starting with 100% air, the air content of the gas mixture was stepwise decreased over the course of the evolution, until the cultures were sparged with 100% nitrogen (< 5 ppm O_2_). The CO_2_ concentration in the off gas was continuously measured to monitor sugar consumption and growth. A sharp decrease in off-gas CO_2_ concentration was interpreted as an indication that exponential growth had ceased. When the concentration of CO_2_ in the off-gas dropped by at least 0.3% below the highest CO_2_ concentration observed (‘the peak’) in that batch, an empty–refill cycle was automatically triggered. To prevent automatic triggering of empty–refill cycles due to noise in CO_2_ measurements in the early phase of the batch, the empty–refill cycle was only triggered after the off-gas CO_2_ concentration of that batch cycle had reached a value of at least 0.5%.

All other bioreactor cultivations were conducted in 2 L laboratory bioreactors (Applikon, Delft, the Netherlands) with a 1 L working volume. Cultures were stirred at 800 rpm, the temperature was controlled at 30 °C and the pH was kept constant at 5.0 through automated addition of 2.0 M KOH. Anaerobic cultures were sparged with 500 mL N_2_ min^−1^ (< 5 ppm O_2_), and medium vessels were sparged with nitrogen as well. IMZ783 and IMZ784 were evolved in SM with 20 g L^−1^ sucrose in an SBR setup. During the evolution, the CO_2_ concentration in the off gas was continuously measured using a MultiExact 4100 analyser (Servomex, Egham, Surrey, United Kingdom) to monitor growth. The end of the batch phase automatically triggered an empty–refill cycle when, after at least 8 h, the CO_2_ concentration in the off gas had decreased to 50% of the maximum CO_2_ concentration of that batch. Cultures were sparged with 500 mL min^−1^ of a gas mixture of air and nitrogen. To be able to control the gas composition, compressed air at 3 bar overpressure was led through a mass flow controller, before connection to tubing with nitrogen at 2 bar. A second mass flow controller after this connection allowed for the control of the flowrate of the gas mixture. Starting with 100% air, the air content of the gas mixture was stepwise decreased over the course of the evolution, until the cultures were sparged with 100% nitrogen and anaerobic conditions were attained. For chemostat cultivations, medium pumps were switched on after sugar depletion in a preceding batch phase, to obtain a constant flowrate. The volume was kept constant at 1 L using an effluent pump that was controlled by an electric level sensor, resulting in a stable dilution rate. Chemostat cultures were assumed to be in steady state when, after five volume changes, the culture dry weight, extracellular concentrations of sugar, ethanol and glycerol and CO_2_ production rate varied by less than 2% over at least 2 more volume changes.

### Analytical methods

Optical density (OD) of yeast cultures was measured at 660 nm using a Libra S11 spectrophotometer (Biochrom, Cambridge, United Kingdom). For dry weight measurements, 10 or 20 mL culture samples (depending on culture density) were filtered on a pre-weighed nitrocellulose filter with pore size 0.45 μm. Subsequently, the filters were washed with demineralized water and dried for 20 min in a microwave oven at 360 W, after which they were weighed again. Concentrations of sugars, ethanol, glycerol and organic acids were measured via HPLC analysis on an Agilent 1260 HPLC, equipped with a Bio-Rad HPX 87H column. Detection was performed by means of an Agilent refractive index detector and an Agilent 1260 VWD detector.

The carbon recovery was calculated as the percentage of the carbon per liter of medium that could be traced back per liter of culture in CO_2_, biomass and products that were measured via HPLC analysis of the culture supernatant. The ‘ingoing’ carbon, in the medium, was calculated from the concentrations of carbon source (glucose, fructose or sucrose) and ethanol (present due to supplementation of Tween 80 and ergosterol) in the medium, which were also determined via HPLC analysis. For the recovered carbon, concentrations of glucose, fructose, glycerol, ethanol, succinate, pyruvate, lactate, acetate and formate were determined in the culture supernatant via HPLC analysis. The amount ethanol that evaporated per liter of culture was estimated using a previously determined volume-dependent ethanol evaporation constant [80]. The amount of CO_2_ produced per liter of culture was calculated by multiplying of the fraction of CO_2_ in the reactor off-gas with the flowrate of the reactor off-gas and the dilution rate, and subsequently dividing by the reactor volume. The flowrate of outgoing, spent media was assumed to be identical to the determined inflow rate. Finally, to calculate the amount of carbon in biomass from dry weight measurements, the composition of 1 Cmol of biomass was assumed to be CH_1.8_O_0.5_N_0.2_, and thus a molar weight of 24.6 g Cmol biomass^−1^ was assumed.

### Structural modeling of KlFrt1 and KmHgt1 proteins

Homology modeling of both KlFrt1 and KmHgt1 was performed using the SWISS-MODEL server [81]. For KlFrt1, the structure of *Escherichia coli* XylE (PDB 4GBY.1.A) was used as template to predict the structure, whereas for modeling of KmHgt1, the structure of *Arabidopsis thaliana* STP10 (PDB 7AAQ.1.A) was used.

## Supplementary Information

**Additional file 1:** **S1.**Theoretical analysis of impact of decreased ATP yield on ethanol yield. **S2. **Table S1: Primers used in this study. **S3. **Table S2: Mutations found in strains evolved for anaerobic growth via whole genome sequencing.

**Additional file 2:** Evolution of hexose proton symporter-expressing strains for anaerobic growth.

## Data Availability

All data generated or analyzed during this study are included in this published article and its supplementary information files.
