# Supplementary material for: Engineering proton-coupled hexose uptake in Saccharomyces cerevisiae for improved ethanol yield
Source: Biotechnol Biofuels Bioprod. 2022 May 7;15:47. doi: 10.1186/s13068-022-02145-7 (PMC9077909; doi:10.1186/s13068-022-02145-7)
Supplement: Supplementary file 1 — Additional file 1: S1.Theoretical analysis of impact of decreased ATP yield on ethanol yield. S2. Table S1: Primers used in this study. S3. Table S2: Mutations found in strains evolved for anaerobic growth via whole genome sequencing. [file 13068_2022_2145_MOESM1_ESM.docx]

# Additional file 1

## S1. Theoretical analysis of impact of decreased ATP yield on ethanol yield

When the ethanol yield on sugar of a reference strain is known, a prediction can be made of the ethanol yield of strain that, compared to that reference strain, conserves less ATP per mol of dissimilated substrate, as previously described by Basso et al. (2011). In this study, strains were engineered in which the net ATP yield of dissimilation was decreased, either from 2 to 1 ATP per hexose, or from 4 to 2 ATP per sucrose (which comes down to 2 to 1 ATP per hexose equivalent), by replacing the facilitated diffusion mechanism of hexoses by proton symport. Consequently, for every hexose entering the cell, an additional hexose equivalent should be completely fermented to ethanol to supply the energy required for transport, which results in a 50% decreased biomass yield compared to a strain with a facilitated diffusion mechanism. The ethanol yield of a strain with proton symport mechanism can be predicted according to Equation 1:

Y_EtOH,1ATP_ = 0.5*Y_EtOH,2ATP_ + 0.5*Y_EtOH,max_ (1)

In Equation 1, Y_EtOH,1ATP_ is the predicted ethanol yield for strains that exclusively take up hexoses by proton symport, Y_EtOH,2ATP_ the measured ethanol yield for a reference strain that takes up hexoses via facilitated diffusion (both in mol ethanol mol hexose equivalent^-1^) and Y_EtOH,max_ the ethanol yield that is obtained upon complete dissimilation via alcoholic fermentation, in this case 2 mol ethanol mol hexose equivalent^-1^.

## S2. Table S1: Primers used in this study

| Primer name | Sequence (5’-3’) |
| --- | --- |
| 1738 | GCAGGCAAGATAAACGAAGG |
| 1742 | GGTCGCCTGACGCATATACC |
| 1743 | TAAGGCCGTTTCTGACAGAG |
| 2873 | TCAGACTTCTTAACTCCTGTAAAAACAAAAAAAAAAAAAGGCATAGCAATAAGCTGGAGCTCATAGCTTC |
| 3093 | ACTATATGTGAAGGCATGGCTATGGCACGGCAGACATTCCGCCAGATCATCAATAGGCACCTTCGTACGCTGCAGGTCGAC |
| 3755 | CACTTGTTCGCTCAGTTCAG |
| 4653 | GTGCCTATTGATGATCTGGCGGAATGTCTGCCGTGCCATAGCCATGCCTTCACATATAGTCCGCAAATTAAAGCCTTCGAG |
| 5542 | CTATGCTACAACATTCCAAAATTTGTCCCAAAAAGTCTTTGGTTCATGATCTTCCCATACGCATAGGCCACTAGTGGATCTG |
| 6005 | GATCATTTATCTTTCACTGCGGAGAAG |
| 6486 | TTTTAGTTTATGTATGTGTTTTTTGTAGTTATAGATTTAAGCAAG |
| 7812 | TCATGTAATTAGTTATGTCACGCTTACATTC |
| 7999 | GGATCCACTAGTTCTAGAAAACTTAGATTAG |
| 9355 | TGTAAATATCTAGGAAATACACTTGTGTATACTTCTCGCTTTTCTTTTATTTTTTTTTGTAGTTTATCATTATCAATACTCGCCATTTC |
| 9356 | TTTACAATATAGTGATAATCGTGGACTAGAGCAAGATTTCAAATAAGTAACAGCAGCAAAGTGTGGAAGAACGATTACAACAG |
| 9522 | CCTACTTTTTTCCGAACATCTTCTTGTAAAATGGTTGATCATCATGCATTAGATCATCAGTCTTTCTTTACCTCTAATATATTCTTTTTTCTAGAAAAATAATATTTTTTGCTGGTTTTA |
| 9523 | TAAAACCAGCAAAAAATATTATTTTTCTAGAAAAAAGAATATATTAGAGGTAAAGAAAGACTGATGATCTAATGCATGATGATCAACCATTTTACAAGAAGATGTTCGGAAAAAAGTAGG |
| 9525 | AGCGGGATACAGAAAAAGAAGATATTTCCCATTTCAAAAAGGCTCTACTATATCCTTACCAAGACTCTAGGGGGATCGCC |
| 9526 | GGTCATTATACTGACCGCCATTATAATGACTGTACAACGACCTTCTGGAGAAAGAAACAAGCGCCTCGTATCTTTTAATG |
| 9528 | TTACTATCAAGATACCGTAGAAAAGAAAAAGAACCGGGGATGAATAATAACAAAACGGGCGAGATTATACTTAAACTAGCACTGATTTTTTTAAGGCTAATGGCTACTAATACTTTAATA |
| 9529 | TATTAAAGTATTAGTAGCCATTAGCCTTAAAAAAATCAGTGCTAGTTTAAGTATAATCTCGCCCGTTTTGTTATTATTCATCCCCGGTTCTTTTTCTTTTCTACGGTATCTTGATAGTAA |
| 9532 | TATTTTAGATTGCATTTTTTATAAGTCACTTTTAGTTAGCTCAGAACGCCAGCAAAAACCCCCGTCAGGACTACCAAAATATAACAAAGTATTGATTATCGTCAACGCTTTATAGATCAC |
| 9533 | GTGATCTATAAAGCGTTGACGATAATCAATACTTTGTTATATTTTGGTAGTCCTGACGGGGGTTTTTGCTGGCGTTCTGAGCTAACTAAAAGTGACTTATAAAAAATGCAATCTAAAATA |
| 9535 | TTGGAAAAAATAATCATTGCACAATTGAGTACTAAAAGCTTTCGTATCTTACCCAATATCTCTTCCGGTTTTTAGTAACTGGAAAAAAATACATGAACTTAATTATTTATTATATACAAT |
| 9536 | ATTGTATATAATAAATAATTAAGTTCATGTATTTTTTTCCAGTTACTAAAAACCGGAAGAGATATTGGGTAAGATACGAAAGCTTTTAGTACTCAATTGTGCAATGATTATTTTTTCCAA |
| 9538 | TCATCAATTTCGGCTGTCTGGATGGCGACAACGAGAGGAAGCTTGCTTGCCATGATAGGAATCAGGCAATGTAAGGTCAAACTTGTACCATCGACATATATAATGCTTTGAGATATAAGT |
| 9539 | ACTTATATCTCAAAGCATTATATATGTCGATGGTACAAGTTTGACCTTACATTGCCTGATTCCTATCATGGCAAGCAAGCTTCCTCTCGTTGTCGCCATCCAGACAGCCGAAATTGATGA |
| 9542 | TTACATAATCATTTCTCCGTGCGAGTTATAAGTGCTTTTCTCGTGCATCCCTTGGCACTTTCTTCTAGTTTTCGGTAAATTGGTAAAAAAGCAAAAAAAAAAATTGACGAAACTAATTCT |
| 9543 | AGAATTAGTTTCGTCAATTTTTTTTTTTGCTTTTTTACCAATTTACCGAAAACTAGAAGAAAGTGCCAAGGGATGCACGAGAAAAGCACTTATAACTCGCACGGAGAAATGATTATGTAA |
| 9547 | CCTTTTACCAATTTAATAATGTCTAGGATTTATCGCTGTACTGCCAAATGCTTCACAACGGAGTGCATATGTTTGTCTTGATAGGCAACATTGTATATTATAGTTTACATAATAATGTGT |
| 9548 | ACACATTATTATGTAAACTATAATATACAATGTTGCCTATCAAGACAAACATATGCACTCCGTTGTGAAGCATTTGGCAGTACAGCGATAAATCCTAGACATTATTAAATTGGTAAAAGG |
| 9551 | ACTAACAAAGAATTTGTTAACGTATTCTTAGGAAGTAAAGTACTTCAATTATTGCTAGAACAGTAACTAAGTGGCCGTGAGGACTGACCACATTTTCTTTACATACAGTAAAGTAAATAA |
| 9552 | TTATTTACTTTACTGTATGTAAAGAAAATGTGGTCAGTCCTCACGGCCACTTAGTTACTGTTCTAGCAATAATTGAAGTACTTTACTTCCTAAGAATACGTTAACAAATTCTTTGTTAGT |
| 9555 | GATTCCACCTTAAAAGACGACAATAGTAACTTTGTCCTTGATCTGGGTTACTAAATCAGCACATAGGTCCACTTGAGTGCTTATGGAAGATCCTTAAAAATGCATTTCCAGGAACGTAAC |
| 9556 | GTTACGTTCCTGGAAATGCATTTTTAAGGATCTTCCATAAGCACTCAAGTGGACCTATGTGCTGATTTAGTAACCCAGATCAAGGACAAAGTTACTATTGTCGTCTTTTAAGGTGGAATC |
| 9563 | GGATTGAAAATTTGGTGTTGTGAATTGCTCTTCATTATGCACCTTATTCAATTATCATCAGATAACATGCTCTGCCATCCTTTGTTCACCGAGCAAAATTAAAAACGCAAAATGAATTGT |
| 9564 | ACAATTCATTTTGCGTTTTTAATTTTGCTCGGTGAACAAAGGATGGCAGAGCATGTTATCTGATGATAATTGAATAAGGTGCATAATGAAGAGCAATTCACAACACCAAATTTTCAATCC |
| 9719 | TCAAGAACTTGTCATTTGTATAG |
| 10305 | GGTTAATTGCGCGCTTGGCGTAATCATGGTCATAGCTGTTTAGTGTGAGCGGGATTTAAACTGTGAGG |
| 10306 | GTTGTGTGGAATTGTGAGCGGATAACAATTTCACACAGGACAGTATAGCGACCAGCATTC |
| 10307 | AACAGCTATGACCATGATTA |
| 10308 | TCCTGTGTGAAATTGTTATC |
| 10519 | TGCGCATGTTTCGGCGTTCGAAACTTCTCCGCAGTGAAAGATAAATGATCTTTTCTTGAAAGCTTTGCAGGTTTTAGAGCTAGAAATAGCAAGTTAAAATAAGGCTAGTCCGTTATCAAC |
| 10521 | AATGTGATTTCTTCGAAGAATATACTAAAAAATGAGCAGGCAAGATAAACGAAGGCAAAGTGACACCGATTATTTAAAGCTGCAGCATACGATATATATACATGTGTATATATGTATACC |
| 10522 | GGTATACATATATACACATGTATATATATCGTATGCTGCAGCTTTAAATAATCGGTGTCACTTTGCCTTCGTTTATCTTGCCTGCTCATTTTTTAGTATATTCTTCGAAGAAATCACATT |
| 11826 | TGCGCATGTTTCGGCGTTCGAAACTTCTCCGCAGTGAAAGATAAATGATCTATCAACAAAATACTCCAATGTTTTAGAGCTAGAAATAGCAAGTTAAAATAAG |
| 12743 | TGCGCATGTTTCGGCGTTCGAAACTTCTCCGCAGTGAAAGATAAATGATCACCGGATTCAGTCGTCACTCAGTTTTAGAGCTAGAAATAGCAAGTTAAAATAAG |
| 13617 | CTAGCATACAAGTTAGAATAAATAAAAAATAGAAAAATAGAACATAGAAAGTTTTAGACCTTCATACACAACTCACAAATCATATTTTGTAATTTACTTAAGAAAGCATTTTGCGGGGTT |
| 13618 | AACCCCGCAAAATGCTTTCTTAAGTAAATTACAAAATATGATTTGTGAGTTGTGTATGAAGGTCTAAAACTTTCTATGTTCTATTTTTCTATTTTTTATTTATTCTAACTTGTATGCTAG |
| 15985 | CTTGCTCATTAGAAAGAAAGCATAGCAATCTAATCTAAGTTTTCTAGAACTAGTGGATCCATGTCTAGTAATCTGTCTGAAA |
| 15986 | CGGTTAGAGCGGATGTGGGGGGAGGGCGTGAATGTAAGCGTGACATAACTAATTACATGATTAAATAGATTTACGGTTACCAG |
| 15987 | CTTGCTCATTAGAAAGAAAGCATAGCAATCTAATCTAAGTTTTCTAGAACTAGTGGATCCATGTCACATGTTAACGCGTCGT |
| 15988 | CGGTTAGAGCGGATGTGGGGGGAGGGCGTGAATGTAAGCGTGACATAACTAATTACATGATTAATAACTCAATTGGCCCTTCTTC |
| 16387 | CTTGCTCATTAGAAAGAAAGCATAGCAATCTAATCTAAGTTTTCTAGAACTAGTGGATCCATGTCGGAACTTGAAAACGC |
| 16388 | CGGTTAGAGCGGATGTGGGGGGAGGGCGTGAATGTAAGCGTGACATAACTAATTACATGATTCTACAAGAGATTATTTTTCTTTAGTG |
| 16508 | CTTGCTCATTAGAAAGAAAGCATAGCAATCTAATCTAAGTTTTCTAGAACTAGTGGATCCATGTCATTGAAAGACAAGATTTTG |
| 16509 | CGGTTAGAGCGGATGTGGGGGGAGGGCGTGAATGTAAGCGTGACATAACTAATTACATGATTAGTTAGAGTTTGAGTTTGAGTTG |
| 17347 | TTAAATCTATAACTACAAAAAACACATACATAAACTAAAAATGTCACATGTTAACGCGTCGT |
| 17348 | ACTACAATATAAAAAAACTATACAAATGACAAGTTCTTGATGATTAATAACTCAATTGGCCCTTCTTC |
| 17349 | TTAAATCTATAACTACAAAAAACACATACATAAACTAAAAATGTCTAGTAATCTGTCTGAAA |
| 17350 | ACTACAATATAAAAAAACTATACAAATGACAAGTTCTTGATTAAATAGATTTACGGTTACCAG |
| 17584 | ACACCAGAACTTAGTTTCGACGGATTCTAGAACTAGTAATATGCTTTTGCAAGCTTTCCTTTTCCTTTTG |
| 17585 | CTTTAAGAATGCCTTAGTCATGCCAACCCTCGAGGTCGACCTATTTTACTTCCCTTACTTGGAACTTGTCAATG |
| 17586 | ATTACTAGTTCTAGAATCCGTCGAAACTAAGTTC |
| 17587 | GTCGACCTCGAGGGTTGG |

## S3. Table S2: Mutations found in strains evolved for anaerobic growth via whole genome sequencing

| **Strain** | **Mutated gene or region** | **Nucleotide change** | **Amino acid change** | **Gene annotation** |
| --- | --- | --- | --- | --- |
| IMS1058 (*MAL11*) | *YPR202w* | G653A | S218N | Putative protein of unknown function; similar to telomere-encoded helicases |
|  | *SED4* | A1457AAAG | F486FS (insertion) | Integral ER membrane protein that stimulates Sar1p GTPase activity |
|  | *SWR1* | G1287T | R429S | Swi2/Snf2-related ATPase; catalytic subunit of SWR1 complex |
|  | *RGT2* | G2191A | D731N | Plasma membrane glucose sensor that regulates glucose transport |
|  | *HRK1* | G315GTGT | S105SS (insertion) | Protein kinase; implicated in activation of the plasma membrane H(+)-ATPase Pma1p in response to glucose metabolism |
| IMS1059  (*KlFRT1*) | *KlFRT1* | T767C | L256S | Fructose proton symporter |
|  | *ECM21* | G972GT (frameshift) | N.A. | Alpha-arrestin, ubiquitin ligase adaptor for Rsp5p; regulates starvation- and substrate-induced Ub-dependent endocytosis of select plasma membrane localized amino acid transporters |
|  | *ATG1* | C1130T | A377V | Protein serine/threonine kinase; required for vesicle formation in autophagy and the cytoplasm-to-vacuole targeting (Cvt) pathway |
|  | Duplication of Chr. I |  |  |  |
| IMS1060  (*SeFSY1*) | *MAL23* | C935T  C936A  C938G  A939C | T312I  T313S | Maltose-responsive transcription factor |
|  | *DTD1* | C436T | L146F | D-Tyr-tRNA(Tyr) deacetylase |
|  | *NFT1* | C1591G | L531V | Putative transporter of the MRP family |
|  | *WHI2* | A861AG (frameshift) | N.A. | Negative regulator of TORC1 in response to limiting leucine |
|  | Duplication of Chr. VIII | N.A. | N.A. | N.A. |
| IMS1061  (*KmHGT1*) | *DOS2* | G903 GGACGACGACGAC | K301KDDDD (insertion) | Protein of unknown function; (GFP)-fusion protein localizes to the cytoplasm |
| IMS1214 (*KmHGT1* + *SeFSY1*) | *KmHGT1* | T1058C | F353S | Glucose proton symporter |
|  | *VAC8* | C1190T | S397F | Vacuole-specific Myo2p receptor and Myo2p-Vac17p-Vac8p transport complex subunit required for vacuolar inheritance |
|  | *PSK2* | G3019A | V1007I | PAS-domain containing serine/threonine protein kinase; regulates sugar flux and translation in response to an unknown metabolite. |
| IMS1215 (*KmHGT1* + *SeFSY1*) | *KmHGT1* | T1058C | F353S | Glucose proton symporter |
|  | *SED4* | T1331A | Q444L | Integral ER membrane protein that stimulates Sar1p GTPase activity |
